# Two Loci on Chromosome 5 Are Associated with Serum IgE Levels in Labrador Retrievers

**DOI:** 10.1371/journal.pone.0039176

**Published:** 2012-06-15

**Authors:** Marta Owczarek-Lipska, Béatrice Lauber, Vivianne Molitor, Sabrina Meury, Marcin Kierczak, Katarina Tengvall, Matthew T. Webster, Vidhya Jagannathan, Yvette Schlotter, Ton Willemse, Anke Hendricks, Kerstin Bergvall, Åke Hedhammar, Göran Andersson, Kerstin Lindblad-Toh, Claude Favrot, Petra Roosje, Eliane Marti, Tosso Leeb

**Affiliations:** 1 Institute of Genetics, Vetsuisse Faculty, University of Bern, Bern, Switzerland; 2 DermFocus, Vetsuisse Faculty, University of Bern, Bern, Switzerland; 3 Department of Clinical Research and Veterinary Public Health, Vetsuisse Faculty, University of Bern, Bern, Switzerland; 4 Department of Clinical Veterinary Medicine, Division of Clinical Dermatology, Vetsuisse Faculty, University of Bern, Bern, Switzerland; 5 Clinic for Small Animal Internal Medicine, Dermatology Unit, Vetsuisse Faculty, University of Zurich, Zurich, Switzerland; 6 Computational Genetics Group, Department of Clinical Sciences, Swedish University of Agricultural Sciences, Uppsala, Sweden; 7 Science for Life Laboratory, Department of Medical Biochemistry and Microbiology, Uppsala University, Uppsala, Sweden; 8 Department of Clinical Sciences of Companion Animals, Faculty of Veterinary Medicine, Utrecht University, Utrecht, The Netherlands; 9 Department of Veterinary Clinical Sciences, Royal Veterinary College, London, United Kingdom; 10 Department of Clinical Sciences, Swedish University of Agricultural Sciences, Uppsala, Sweden; National Cancer Institute, United States of America

## Abstract

Crosslinking of immunoglobulin E antibodies (IgE) bound at the surface of mast cells and subsequent mediator release is considered the most important trigger for allergic reactions. Therefore, the genetic control of IgE levels is studied in the context of allergic diseases, such as asthma, atopic rhinitis, or atopic dermatitis (AD). We performed genome-wide association studies in 161 Labrador Retrievers with regard to total and allergen-specific immunoglobulin E (IgE) levels. We identified a genome-wide significant association on CFA 5 with the antigen-specific IgE responsiveness to *Acarus siro*. We detected a second genome-wide significant association with respect to the antigen-specific IgE responsiveness to *Tyrophagus putrescentiae* at a different locus on chromosome 5. *A. siro* and *T. putrescentiae* both belong to the family *Acaridae* and represent so-called storage or forage mites. These forage mites are discussed as major allergen sources in canine AD. No obvious candidate gene for the regulation of IgE levels is located under the two association signals. Therefore our studies offer a chance of identifying a novel mechanism controlling the host's IgE response.

## Introduction

Immunoglobulin E (IgE) is the class of antibodies that is most frequently recognized for its role in type I hypersensitivity (allergic) reactions. In predisposed, atopic individuals IgE is produced against specific common environmental antigens. Most of the IgE is bound on the surface of mast cells through the high affinity IgE receptor (FcεRI). Cross-linking of mast-cell bound IgEs by allergens leads to the release of histamine and many other mediators, and subsequently to allergic reactions in the skin, respiratory tract, or other organs [1]–[3].

Total or allergen-specific IgE levels have been analyzed as correlated endophenotypes for different allergic diseases, such as asthma, atopic rhinitis, or atopic dermatitis (AD). IgE levels were analyzed instead of directly using the disease status, as they show a higher heritability than the disease status and are assumed to be less susceptible to confounding environmental factors [4], [5]. The heritability of total serum IgE levels in humans was estimated to be up to 80% [6], [7]. In humans, several genome-wide association studies (GWAS) were performed to search for QTL with an influence on IgE levels [5], [8]–[10]. So far, about five IgE QTL have been identified by this approach. These loci comprise several functional candidate genes such as e.g. the gene encoding the FcεRIα subunit of the high affinity receptor for IgE (*FCER1A*) on HSA 1q23 or the *IL13, IL4, RAD50* region of HSA 5q31 [3]. The causative mutations underlying these QTL are not yet known.

Dogs are valuable models for many human diseases and the special population structure of purebred dogs greatly facilitates the identification of genetic risk factors [11]. AD occurs in humans and dogs [12]–[14] and there are certain dog breeds, which are genetically predisposed to develop AD [15]–[18]. IgE levels in dogs are routinely analyzed to aid in the diagnosis of canine AD. In the last years, it has been recognized that total serum IgE levels do not correlate with AD status in dogs [19]. The level of total IgE in dogs is probably much more influenced by the load of endoparasites than by responses to environmental allergens [20]. However, findings of elevated allergen-specific serum IgE levels are used together with clinical criteria to diagnose canine AD. Allergen-specific serum IgE levels are thus indicative but not pathognomonic for AD as healthy dogs may also show elevated allergen-specific IgE levels [19], [21].

The major recognized environmental allergens appear to be similar between humans and dogs and include house dust and storage mites, pollens, moulds, and insects [22]. In temperate climates the house dust mites of the genus *Dermatophagoides* and especially *D. farinae* are considered the most important sources of allergen in humans and dogs and several allergens have been identified on a molecular level [23]. In addition to the “true” house dust mites, most dogs are also exposed to storage or forage mites, most notably *Tyrophagus putrescentiae*, *Acarus siro*, and *Lepidoglyphus destructor*. It has been shown that such mites can be present even in un-opened bags of dry dog food. Under suitable environmental conditions up to 90% of commercial dry foods became contaminated with forage mites within a couple of weeks after opening of the bags [24]. As exposure to forage mites seems to be quite universal in human dwellings and the term “domestic mites” has been suggested for the whole group of house dust and forage mites [25].

We are currently building the LUPA cohort of Labrador Retrievers to gain a better understanding of IgE regulation and its relation to AD. We report here the results of a GWAS with respect to immunological traits in this cohort.

## Results

We collected sera from 161 Labrador Retrievers and determined 15 immunological phenotypes consisting of *Dermatophagoides farinae* specific IgG1 and IgG4, total IgE, and 12 additional allergen-specific IgE serum levels against *Dermatophagoides farinae*, *Dermatophagoides pteronyssinus*, *Tyrophagus putrescentiae*, *Lepidoglyphus destructor*, *Acarus siro*, *Alternaria alternata*, *Cladosporium herbarum*, *Aspergillus fumigatus*, *Penicillinum sp*, cat epithelium, flea saliva, and *Blatella germanica*.

*A. siro* is a storage mite considered to be a possible source of antigens that may provoke AD. We determined the *A. siro*-specific serum IgE levels by an enzyme-linked immunosorbent assay (ELISA) and expressed them in ELISA units (EU). The measured IgE levels ranged from 0 to 3060 EU. The lower reliable detection limit for the *A. siro*-specific IgE level is 150 EU. We treated the IgE levels as a binary trait and considered dogs with more than 150 EU as IgE-responsive to *A. siro* and dogs with less than 150 EU as IgE-negative controls.

We determined the genotypes of 174,376 SNP markers in all individuals. Based on genetic distances between individuals projected into two-dimensional space using multidimensional scaling we excluded three dogs that were outliers, so that 135 *A. siro*-specific IgE responders (cases) and 24 controls remained for the final analysis (Figure S1). We also excluded non-informative markers and markers with low call rates and had 113,021 SNP markers for the final analysis.

**Figure 1:**
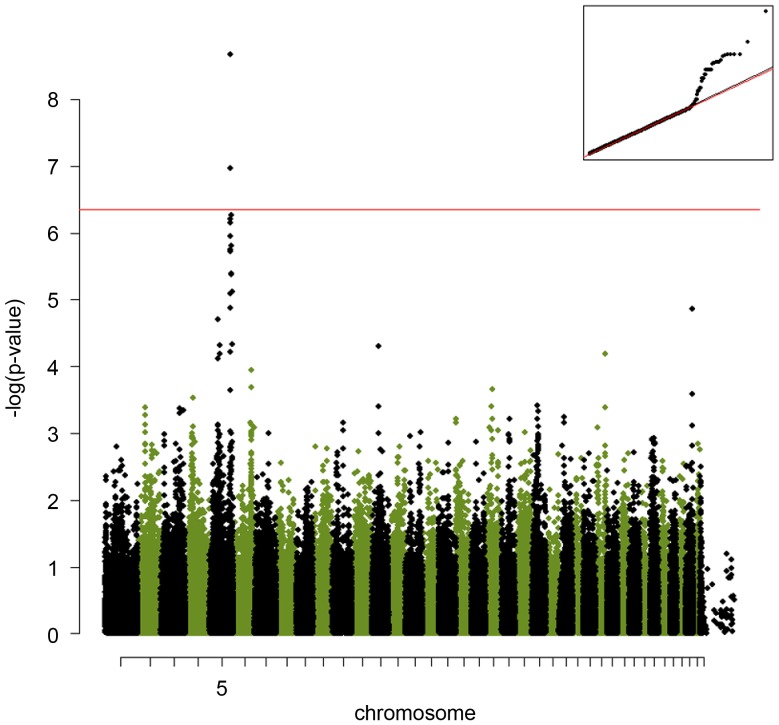
A genome-wide association study of the IgE-responsiveness to *A. siro* in Labrador Retrievers performed using a mix-model approach efficiently corrected for the population stratification. The red line indicates the Bonferroni-corrected significance level (p<3.9×10^−7^). The Quantile-quantile (QQ) plot shows the observed versus expected log p-values (on the top-right). The straight line on the QQ plot indicates the distribution of SNP markers under the null hypothesis and the skew at the right edge indicates that these markers are stronger associated with the *A. siro* IgE response than it would be expected by chance.

For the selection of dogs we had minimized the use of first-degree relatives in order to reduce the stratification of our samples. Nonetheless, in purebred dogs there is always a certain amount of cryptic relatedness present. A preliminary analysis without correction for cryptic relatedness gave a genomic inflation factor of 1.09 indicating a relatively low level of stratification. For the GWAS we used a mixed model approach using the marker-derived kinship matrix as a co-variable to correct for both stratification and cryptic relatedness. After this correction the genomic inflation factor was 1.00. We performed an allelic association analysis and detected one region on chromosome 5 (CFA 5) that was significantly associated with the IgE response to *A. siro*. The best-associated SNPs within the interval, BICF2S2297212 (CFA5:g.79,522,993A>G), showed a raw p-value of 2.14×10^−9^. This is below the Bonferroni-corrected significance threshold of 4.4×10^−7^. We also determined the empirical significance threshold by performing 100,000 permutations with randomly assigned phenotypes, which yielded a genome-wide corrected p-value of 0.001 (Figure 1, Table 1). A second marker at the same locus (BICF2P1022237; CFA5:g.80,037,053C>T) was also significantly associated with the *A. siro*-specific IgE response with a raw p-value of 1.07×10^−7^. Apart from these two SNPs, no other SNP on any chromosome reached genome-wide significance.

**Table 1:** Top allelic association hits in the GWAS for the IgE responsiveness to *A. siro* on CFA5.

SNP	Position	Alleles	Allele frequencies (136 cases/25 controls)	p_raw_ a
BICF2S2297212	79,522,993	G/A	0.04/0.33	2.14×10^−9^
BICF2P1022237	80,037,053	C/T	0.05/0.33	1.07×10^−7^
BICF2P1226027	81,633,676	G/A	0.03/0.24	5.36×10^−7^
BICF2P1359501	81,697,025	G/A	0.03/0.24	5.36×10^−7^
BICF2P501959	81,740,735	T/C	0.03/0.24	5.36×10^−7^
BICF2P1096337	81,762,550	C/T	0.03/0.24	5.36×10^−7^
BICF2P373634	80,027,563	C/T	0.03/0.26	6.20×10^−7^
BICF2P641629	79,668,045	C/T	0.04/0.28	7.13×10^−7^
BICF2P1263867	78,984,517	C/T	0.04/0.30	1.12×10^−6^
BICF2P526058	81,916,847	A/G	0.03/0.24	1.57×10^−6^
BICF2P570236	81,927,096	C/T	0.03/0.24	1.57×10^−6^
BICF2P989928	81,950,340	G/A	0.03/0.24	1.57×10^−6^
BICF2P120791	79,047,969	T/G	0.04/0.26	1.75×10^−6^
BICF2P703501	79,079,463	G/A	0.04/0.26	1.75×10^−6^
BICF2P523308	79,008,866	T/G	0.04/0.26	1.91×10^−6^
BICF2P159065	82,120,219	G/A	0.04/0.26	3.98×10^−6^
BICF2S23528772	82,134,285	C/T	0.04/0.26	3.98×10^−6^
BICF2P318446	81,863,636	G/A	0.04/0.26	4.02×10^−6^
BICF2P139695	81,869,371	A/G	0.04/0.26	4.02×10^−6^
BICF2P1431	81,885,244	C/T	0.04/0.26	4.02×10^−6^
BICF2P856483	81,892,362	C/T	0.04/0.26	4.02×10^−6^
TIGRP2P74638	82,033,585	G/A	0.04/0.24	4.15×10^−6^
BICF2P323747	86,660,476	C/A	0.03/0.17	7.61×10^−6^
BICF2P1319778	81,083,575	A/G	0.04/0.24	8.19×10^−6^
BICF2P1142629	80,190,234	G/A	0.03/0.22	1.30×10^−5^
BICF2P674952	80,213,154	A/G	0.03/0.22	1.30×10^−5^
BICF2P1329549	80,229,734	T/C	0.03/0.22	1.30×10^−5^

a p-values were calculated using χ^2^ tests in an allelic association study.

**Figure 2:**
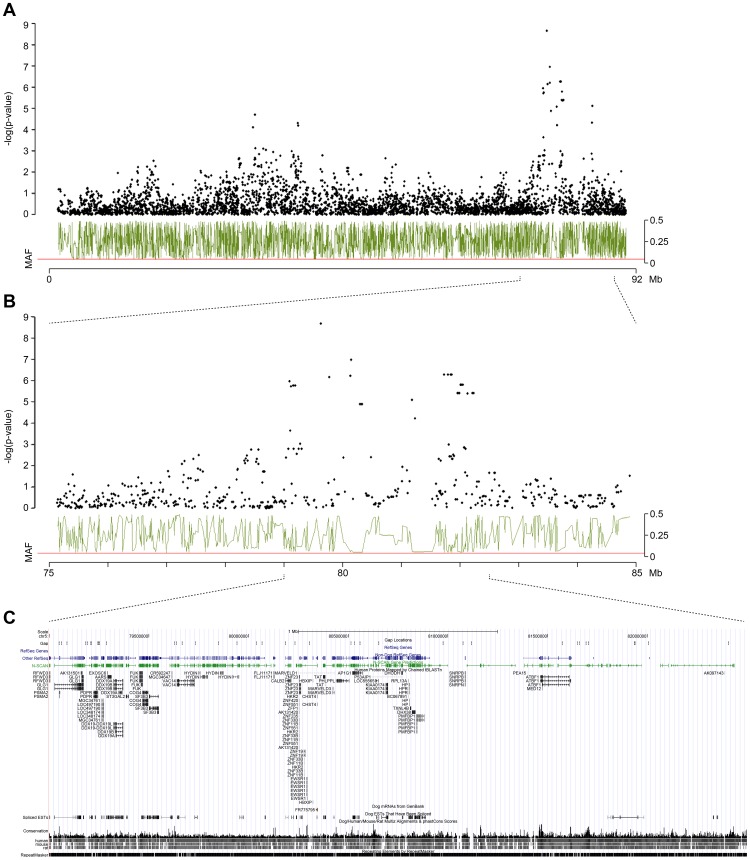
Results of the genome-wide association study restricted to SNP markers located on CFA5. (**A**) GWAS in the cohort of 161 Labradors showed a significant association of the elevated *A. siro* specific IgE levels in 136 *A. siro* IgE-positive dogs and 25 *A. siro* IgE-negative dogs. (**B**) Associated SNP markers (black) and minor allele frequency (green) at the locus of the IgE-responsiveness to *A. siro* zoomed into the interval between 75 Mb and 85 Mb. (**C**) The gene content of the 3.5-Mb chromosomal interval estimated according to the dog build 2 and displayed using UCSC Genome Browser (http://genome.ucsc.edu).

The 27 best associated markers of the GWAS were clustered on CFA 5 in an interval ranging from 79.0–86.7 Mb. Only the 28th best-associated marker resided at a different locus on CFA 37. The observed linkage disequilibrium of the association signals on CFA 5 delineates the associated interval to approximately 3.5 Mb ranging from 79.0–82.5 Mb (Figure 2).

The canine genome annotation still remains imperfect. Therefore, we extrapolated the gene content of the associated region from the orthologous human genome interval. The associated region matches to two counterparts on HSA 16, located between 70.1–74.0 Mb and 74.4–74.7 Mb, respectively (Figure 2). This region is very gene-rich and contains 69 annotated genes in the human genome (NCBI build 37.3). There is no obvious functional candidate gene known to be involved in IgE regulation in the associated interval. However, there are some genes related to the immune system, such as e.g. the interleukin 34 gene (*IL34*), in this interval.

Apart from the association to *A. siro*-specific IgE levels we also found a significantly associated SNP with respect to the allergen-specific IgE levels against *Tyrophagus putrescentiae* (Table 2). The marker BICF2G630182288 at position 33,656,088 on CFA 5 has a raw p-value of 6.10×10^−8^. This marker is 46 Mb away from the highest association to *A. siro*-specific IgE levels. Interestingly however, the region with the *T. putrescentiae*-specific IgE association also showed a moderate association with respect to *A. siro*-specific IgE levels (Table 2 and Figure 2A).

**Table 2:** Genome-wide association results with respect to all studied traits.

Allergen	Responders (cases)	Non-responders (controls)	Chromosome^a^	Best associated SNP	Position	p_raw_	No. of asso-ciated SNPs^b^
***Acarus siro*** ** IgE**	**136**	**25**	**5**	**BICF2S2297212**	**79,522,993**	**2.14×10^−9^**	**8**
***Tyrophagus putrescentiae*** ** IgE**	**145**	**16**	**5**	**BICF2G630182288** c	**33,656,088**	**6.10×10^−8^**	**6**
*Blattela germanica* IgE	10	151	14	BICF2P1257996	62,500,248	9.92×10^−7^	1
*Cat epithelium* IgE	13	148	14	BICF2P1257996	62,500,248	9.92×10^−7^	1
*Aspergillus fumigates* IgE	13	148	14	BICF2P1257996	62,500,248	9.92×10^−7^	1
*Penicillinum sp.* IgE	11	150	27	BICF2P140154	10,490,910	1.30×10^−6^	0
*Flea saliva* IgE	17	144	3	BICF2S23259979	25,656,748	3.31×10^−6^	0
*Dermatophagoides pteronyssinus* IgE	75	86	14	BICF2P471228	62,894,579	8.78×10^−6^	0
Total IgE	93	68	7	BICF2G630551420	14,468,374	1.75×10^−5^	0
*Dermatophagoides farina* IgE	133	15	14	BICF2P45581	37,505,424	3.19×10^−5^	0
*Alternaria alternate* IgE	53	108	6	BICF2S22949377	30,641,636	3.91×10^−5^	0
*Dermatophagoides farinae* IgG1	67	81	12	BICF2P376143	51,541,485	4.02×10^−5^	0
*Lepidoglyphus destructor* IgE	24	137	12	BICF2P376143	51,541,485	5.81×10^−5^	0
*Cladosporium herbarum* IgE	12	149	36	BICF2G630756211	31,175,618	7.09×10^−5^	0
*Dermatophagoides farinae* IgG4	72	76	12	BICF2P376143	51,541,485	7.77×10^−5^	0

Associations exceeding the Bonferroni-corrected significance threshold of p_raw_=4.4×10^−7^ are shown in bold.

a Chromosome with the best associated marker.

b Number of SNPs at the locus with p_raw_<10^−6^. Purebred dogs typically show long-range linkage disequilibrium within breeds. Therefore, strong association signals within dog breeds are often supported by multiple SNPs within a 1–2 Mb interval.

c This SNP had a p-value of 7.66×10^−5^ in the GWAS with respect to *A. siro* specific IgE levels. *T. putrescentiae* and *A. siro* both belong to the group of storage mites.

**Figure 3:**
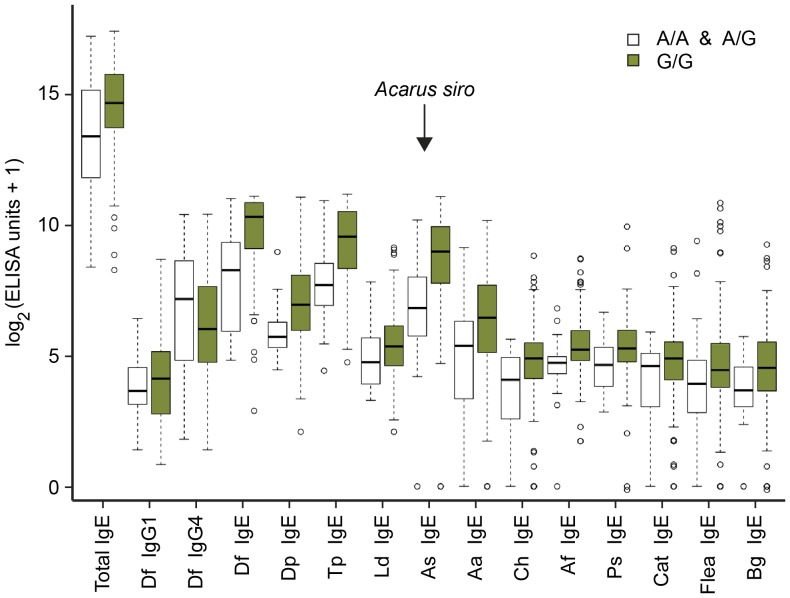
ELISA measurements of 15 immunological phenotypes depending on the genotype at SNP BICF2S2297212. The G-allele at this SNP was associated with the allergen-specific IgE response to *A. siro*. Note that dogs with the homozygous G/G genotype show a consistent trend for higher IgE levels across all measured antigens. Boxes represent the 25^th^ and 75^th^ percentile of the phenotypic distribution with the median indicated by a solid horizontal line. The whiskers indicate the minimum and maximum of the distributions. Outliers that are more than 1.5 times the interquartile range away from the median are shown as open circles. Abbreviations for allergens: Df, *Dermatophagoides farinae*; Dp, *Dermatophagoides pteronyssinus*; Tp, *Tyrophagus putrescentiae*; Ld, *Lepidoglyphus destructor*; As, *Acarus siro*; Aa, *Alternaria alternate*; Ch, *Cladosporium herbarum*; Af, *Aspergillus fumigates*; Ps, *Penicillinum sp.*; Cat, cat epithelium; Flea, flea saliva; Bg, *Blattela germanica*.

We further analyzed whether there are any correlations between the *A. siro*-specific IgE responsiveness and the other studied traits. The best-associated SNP for this trait was BICF2S2297212 (Table 1). The A-allele at this SNP had a frequency of 7.8% and occurred almost exclusively in *A. siro*-specific IgE non-responsive dogs. When we grouped the dogs according to their genotypes at the associated SNP, we observed a consistent trend for dogs with the A-allele to have lower total and lower allergen-specific IgE levels with respect to all tested allergens compared to the dogs that were homozygous G/G. The genotype at this SNP had no visible correlation with the *Dermatophagoides farinae*-specific IgG1 or IgG4 levels (Figure 3).

## Discussion

Atopy is the predisposition to increased IgE production and immediate hypersensitivity in response to exposure to various environmental allergens [26]. Often, intradermal testing (IDT) and/or allergen-specific serological IgE tests are used to support the clinical diagnosis of canine AD. AD is a complex disease influenced by many genetic and environmental factors [16]–[18], [27], [28]. AD affected dogs are often sensitized to the common house dust mite *Dermatophagoides farinae* [29]. However, Roque *et al.* [30] reported controversial findings where significantly higher *Dermatophagoides farinae*-IgE levels were found in non-atopic compared to atopic dogs.

It is well known that *Dermatophagoides farinae* allergens provoke positive immunological reactions in humans and dogs [29]. It has also been reported that storage mites such as *Acarus siro*, *Glycophagus domesticus*, *Tyrophagus putrescentiae*, and *Lepidoglyphus destructor*, which are closely related to house dust mites, are involved in sensitization and allergy induction in humans [31]. To investigate which factors influence allergen-specific IgE levels in Labrador Retrievers we performed GWAS for 12 allergen-specific IgE levels in addition to total IgE and *Dermatophagoides farinae*-specific IgG1, and IgG4 levels as proposed in a human AD study [32]. It has been reported that 18%–50% of atopic dogs manifest sensitization to *A. siro* [29], [33]. We found a significant association of the IgE responsiveness to *A. siro* in Labradors on CFA 5. We performed the GWAS using the allergen-specific IgE levels as a binary trait and we also confirmed these results by treating the IgE levels as continuous trait (data not shown).

The locus of the *A. siro* IgE-responsiveness is located between 79.0 Mb and 82.5 Mb. In the investigated canine chromosomal region no obvious candidate gene for the IgE-responsiveness is located. The interval contains the *IL34* gene encoding a member of the interleukin family, but this gene has so far not been implicated in the regulation of IgE levels [34]. Up till now, the genome region around the *IL34* gene has not been associated with IgE levels in human GWAS [4].

At first glance, it may seem surprising that one locus should influence the allergen-specific IgE levels against *A. siro*, but none of the other allergens. This experimental finding is most likely due to the skewed distributions of IgE responders and non-responders with respect to the different antigens investigated. Our detailed analysis of dogs with “*A. siro* IgE non-responsive” and “*A. siro* IgE responsive” genotypes at the associated SNP revealed a consistent trend for lower IgE but not lower IgG levels across all antigens in the non-responsive genotype class (Figure 3). Thus, the detected locus on CFA 5 may in fact influence the IgE response in general rather than the IgE response to only *A. siro*. Further studies with increased cohort sizes are necessary to conclusively prove this hypothesis.

In conclusion, we mapped two loci for the IgE response to *A. siro* and *T. putrescentiae*, respectively, in Labrador Retrievers. There is no clear functional candidate gene within the associated regions. Thus, this study might eventually lead to the functional annotation of one or several orphan genes and facilitate a better understanding of the immune response to environmental antigens in dogs and humans.

## Materials and Methods

### Ethics statement

All animal experiments were performed according to the local regulations. The dogs in this study were examined with the consent of their owners. The study was approved by the “Cantonal Committee For Animal Experiments” (Canton of Bern; permits 22/07 and 23/10).

### Animal selection

We collected 161 serum and EDTA blood samples from Labrador Retrievers, including 136 dogs with high *A. siro*-specific IgE and 25 dogs with low *A. siro*-specific IgE serum levels (see below). This work was part of a larger effort to build the LUPA AD cohort, which currently consists of 302 Labrador Retrievers. The dogs were clinically diagnosed according to established criteria for canine AD [35], [36] by the clinical dermatology services of the Universities of Bern, London, Uppsala, Utrecht, or Zurich. Control dogs were also investigated by clinical dermatologists. Among dogs with elevated *A. siro*-specific IgE levels 61 were diagnosed as AD-affected and 75 as controls, whereas among dogs with low levels of *A. siro*-specific IgE 16 were AD-affected and 9 were controls. The samples were recruited from 2008–2010.

### DNA samples and SNP genotyping

Genomic DNA samples were isolated from EDTA blood with the Nucleon Bacc2 kit (GE Healthcare). The DNA was genotyped at the Centre National de Génotypage, Evry, France using illumina canine_HD chips containing 174,376 SNP markers. Genotypes were stored in a BC/SNPmax Database version 3.4 (BC/Platforms).

### Measurements of allergen-specific immunoglobulin serum levels

Allergen-specific serum IgE levels against *Dermatophagoides farinae*, *Dermatophagoides pteronyssinus*, *Tyrophagus putrescentiae*, *Lepidoglyphus destructor*, *Acarus siro*, *Alternaria alternata*, *Cladosporium herbarum*, *Aspergillus fumigatus*, *Penicillinum sp*, cat epithelium, flea saliva, and *Blatella germanica* were measured by a commercially available test (Allercept™ IgE Test Panel, Heska AG, Fribourg, Switzerland) [37]. The Heska laboratory was not aware of the clinical diagnosis. The assay was considered as positive when the optical density measured at 405 nm was >150 EU. The threshold was set by the company by taking a large number of sera that tested very low in the assay which were then heated each at 56°C for 4 hours to inactivate IgE and tested again. Each heated serum in this group was defined as negative for allergen specific IgE. The mean EU value plus 3 standard deviations for the group of negative sera was calculated for each of the different allergens, and then an average mean plus 3 SD for all allergens was calculated (150 EU) and used as the cut off.

Total serum IgE levels were also determined by ELISA. Briefly: Plates (Thermo, VWR) were coated with anti-canine IgE monoclonal antibody D9 [38]. Blocking of non-specific binding sites followed, before addition of the test sera, or of purified canine IgE (Bethyl Laboratories, Inc.) used to generate a standard curve on each plate. The canine sera were serially twofold diluted starting at a dilution of 1∶100. After incubation and washing, biotinylated monoclonal anti-canine IgE antibody 5.91 [39] was added for 1 h, followed by extravidine-alkaline phosphatase (Sigma). The plates were then developed with a of phosphatase substrate (Sigma) in diethanolamine (Fluka) solution. Absorbance readings were measured at 405 nm. The values of the test sample were calculated from the standard curve using an ELISA software program (SOFTmax® 2.31 for Windows™, Molecular Devices Co., Sunnyvale, CA, USA).

Relative concentration of the IgG subclasses IgG1 and IgG4, specific for *Dermatophagoides farinae* were determined in the sera of AD and control dogs. 96 well plates (Thermo, VWR) were coated with *Dermatophagoides farinae* extract (Allergomed AG, 4106 Therwil, Switzerland). After blocking, dog sera were serially twofold diluted and added to the plate. A serum pool from dogs immunized with *Dermatophagoides farinae* was used to generate a standard curve on each plate. After incubation and washing, monoclonal antibodies specific for canine IgG1 (clone B6) or IgG4 (clone A5) were added to the plates [40]. An alkaline phosphatase- conjugated affinity purified goat anti-mouse IgG (Jackson ImmunoResearch Inc.) followed. Plates were then developed with phosphatase substrate. Absorbance readings and calculating of IgG1 or IgG4 levels in relative ELISA units were performed as described above.

The *Dermatophagoides farinae* specific IgG1 and IgG4 as well as the total IgE serum levels were transformed into categorical traits by using the median as cut off.

### Genome-wide association studies

We used GenABEL [41] to perform genome-wide association analyses (GWAS). We removed markers with call rates <95% and individuals with call rates <90% from the analysis. For analyses with respect to *Dermatophagoides farinae* specific immunoglobulin levels (IgG1, IgG4, IgE), we removed 13 dogs that had received allergen-specific immunotherapy with *Dermatophagoides farinae* extracts. We also removed markers with minor allele frequency (MAF)<5% and markers strongly deviating from Hardy-Weinberg equilibrium (p<10^−5^). We performed an allelic association study using the h2a2 function, which uses a mixed-model approach, which uses the kinship matrix estimated from the marker data to correct for population stratification. Significance levels were determined by the Bonferroni correction (p_Bonf_=0.05/number of markers). Additionally, empirical significance thresholds were also determined by performing 100,000 permutations of the dataset with arbitrarily assigned phenotypes.

### Gene analysis

We used the dog build 2 and the human build 37 assemblies for all analyses. We used BLASTN searches to define the orthologous human chromosomal regions corresponding to the associated interval on CFA 5. For the candidate gene inspection we used the human annotation provided by NCBI (build 37.3).

## Supporting Information

Figure S1 Multidimensional scaling (MDS) plot showing the genomic kinships between the analyzed Labrador Retrievers. This plot visualizes the overall genetic distances between the dogs based on 2,000 markers randomly selected out of the total of 113,021 SNP markers. Cases and controls do not form separate clusters, which is an essential prerequisite for a successful GWAS.
(TIF)

## References

[1] Gould HJ, Sutton BJ, Beavil AJ, Beavil RL, McCloskey N (2003). The biology of IGE and the basis of allergic disease.. Annu Rev Immunol.

[2] Gould HJ, Sutton BJ (2008). IgE in allergy and asthma today.. Nat Rev Immunol.

[3] Potaczek DP, Kabesch M (2012). Current concepts of IgE regulation and impact of genetic determinants.. Clin Exp Allergy.

[4] Weidinger S, Baurecht H, Naumann A, Novak N (2010). Genome-wide association studies on IgE regulation: are genetics of IgE also genetics of atopic disease?. Curr Opin Allergy Clin Immunol.

[5] Weidinger S, Gieger C, Rodriguez E, Baurecht H, Mempel M (2008). Genome-wide scan on total serum IgE levels identifies FCER1A as novel susceptibility locus.. PLoS Genet.

[6] Hanson B, McGue M, Roitman-Johnson B, Segal NL, Bouchard TJ (1991). Atopic disease and immunoglobulin E in twins reared apart and together.. Am J Hum Genet.

[7] Strachan DP, Wong HJ, Spector TD (2001). Concordance and interrelationship of atopic diseases and markers of allergic sensitization among adult female twins.. J Allergy Clin Immunol.

[8] Moffatt MF, Gut IG, Demenais F, Strachan DP, Bouzigon E (2010). A large-scale, consortium-based genomewide association study of asthma.. N Engl J Med.

[9] Granada M, Wilk JB, Tuzova M, Strachan DP, Weidinger S (2012). A genome-wide association study of plasma total IgE concentrations in the Framingham Heart Study.. J Allergy Clin Immunol.

[10] Ramasamy A, Curjuric I, Coin LJ, Kumar A, McArdle WL (2011). A genome-wide meta-analysis of genetic variants associated with allergic rhinitis and grass sensitization and their interaction with birth order.. J Allergy Clin Immunol.

[11] Karlsson EK, Lindblad-Toh K (2008). Leader of the pack: gene mapping in dogs and other model organisms.. Nat Rev Genet.

[12] Reedy LM, Miller WH, Willemse A (1997). Allergic skin diseases of dogs and cats, 2nd edn.

[13] de Weck AL, Mayer P, Stumper B, Schiessl B, Pickart L (1997). Dog allergy, a model for allergy genetics.. Int Arch Allergy Immunol.

[14] Schlotter YM, Rutten VP, Riemers FM, Knol EF, Willemse T (2011). Lesional skin in atopic dogs shows a mixed Type-1 and Type-2 immune responsiveness.. Vet Immunol Immunopathol.

[15] Griffin CE, DeBoer DJ (2001). The ACVD task force on canine atopic dermatitis (XIV): clinical manifestation of canine atopic dermatitis.. Vet Immunol and Immunopathol.

[16] Jaeger K, Linek M, Power HT, Bettenay SV, Zabel S (2010). Breed and site predisposition of dogs with atopic dermatitis: a comparison of five locations in three continents.. Vet Dermatol.

[17] Sousa CA, Marsella R (2001). The ACVD task force on canine atopic dermatitis (II): genetic factors.. Vet Immunol Immunopathol.

[18] Nødtvedt A, Egenvall A, Bergvall K, Hedhammar A (2006). Incidence of and risk factors for atopic dermatitis in a Swedish population of insured dogs.. Vet Rec.

[19] Halliwell R, DeBoer DJ (2001). The ACVD task force on canine atopic dermatitis (III): the role of antibodies in canine atopic dermatitis.. Vet Immunol Immunopathol.

[20] Jackson HA, Orton SM, Hammerberg B (2002). IgE is present on peripheral blood monocytes and B cells in normal dogs and dogs with atopic dermatitis but there is no correlation with serum IgE concentrations.. Vet Immunol Immunopathol.

[21] DeBoer DJ, Hillier A (2001). The ACVD task force on canine atopic dermatitis (XV): fundamental concepts in clinical diagnosis.. Vet Immunol Immunopathol.

[22] Nuttall TJ, Hill PB, Bensignor E, Willemse T, members of the International Task Force on Canine Atopic Dermatitis (2006). House dust and forage mite allergens and their role in human and canine atopic dermatitis.. Vet Dermatol.

[23] McCall C, Hunter S, Stedman K, Weber E, Hillier A (2001). Characterization and cloning of a major high molecular weight house dust mite allergen (Der f 15) for dogs.. Vet Immunol and Immunopathol.

[24] Brazis P, Serra M, Sellés A, Dethioux F, Biourge V (2008). Evaluation of storage mite contamination of commercial dry dog food.. Vet Dermatol.

[25] Platts-Mills TA, Vervloet D, Thomas WR, Aalberse RC, Chapman MD (1997). Indoor allergens and asthma: report of the Third International Workshop.. J Allergy Clin Immunol.

[26] Jackola DR, Pierson-Mullany LK, Liebeler CL, Blumenthal MN, Rosenberg A (2002). Variable binding affinities for allergen suggest a “selective competition” among immunoglobulins in atopic and non-atopic humans.. Mol Immunol.

[27] Nødtvedt A, Bergvall K, Sallander M, Egenvall A, Emanuelson U, Hedhammar A (2007). A case-control study of risk factors for canine atopic dermatitis among Boxer, Bullterrier and West Highland White Terrier dogs in Sweden.. Vet Dermatol.

[28] Meury S, Molitor V, Doherr MG, Roosje P, Leeb T, Hobi S, Wilhelm S, Favrot C (2011). Role of the environment in the development of canine atopic dermatitis in Labrador and golden retrievers.. Vet Dermatol.

[29] Bensignor E, Carlotti DN (2002). Sensitivity patterns to house dust mites and forage mites in atopic dogs: 150 cases.. Vet Dermatol.

[30] Roque J, O'Leary CA, Kyaw-Tanner M, Latter M, Mason K (2011). High allergen-specific serum immunoglobulin E levels in nonatopic West Hightland white terriers.. Vet Dermatol.

[31] Cookson W (2004). The immunogenetics of asthma and eczema: a new focus on the epithelium.. Nat Rev Immunol.

[32] Jackola DR, Miller MB, Liebeler CL, Blumenthal MN (2007). Search for quantitative trait loci of atopy-associated immune responses using allergen-specific IgG1 as an “endophenotype”. Hum Immunol.

[33] Arlian LG, Schumann RJ, Morgan MS, Glass RL (2003). Serum immunoglobulin E against storage mite allergens in dogs with atopic dermatitis.. Am J Vet Res.

[34] Lin H, Lee E, Hestir K, Leo C, Huang M (2008). Discovery of a cytokine and its receptor by functional screening of the extracellular proteome.. Science.

[35] Willemse T (1986). Atopic skin disease. a review and reconsideration of diagnostic criteria.. J Small Animal Pract.

[36] Prélaud P, Guaguère E, Alhaidari Z, Faive N, Heripret D (1998). Reevaluation of diagnostic criteria of canine atopic dermatitis.. Rev Med Vet.

[37] Lauber B, Molitor V, Meury S, Doherr MG, Favrot C (2012). Total IgE and allergen-specific IgE and IgG antibody levels in sera of atopic dermatitis affected and non-affected Labrador- and Golden retrievers.. Vet Immunol Immunopathol.

[38] DeBoer DJ, Ewing KM, Schultz KT (1993). Production and characterization of mouse monoclonal antibodies directed against canine IgE and IgG.. Vet Immunol Immunopathol.

[39] Hammerberg B, Bevier D, DeBoer DJ, Olivry T, Ortin SM (1997). Auto IgG anti-IgE and IgG X IgE immune complex presence and effects on ELISA -based quantitation of IgE in canine atopic dermatitis, demodectic acariasis and helminthiasis.. Vet Immunol Immunopathol.

[40] Mazza G, Whiting AH, Day MJ, Duffus WP (1994). Development of an enzyme-linked immunosorbent assay for the detection of IgG subclasses in the serum of normal and diseased dogs.. Res Vet Sci.

[41] Aulchenko YS, Ripke S, Isaacs A, van Duijn C (2007). GenABEL: an R library for genome-wide association analysis.. Bioinformatics.

