# Supplementary figures and images for: Two Loci on Chromosome 5 Are Associated with Serum IgE Levels in Labrador Retrievers
Source: PLoS One. 2012 Jun 15;7(6):e39176. doi: 10.1371/journal.pone.0039176 (PMC3376118; doi:10.1371/journal.pone.0039176)

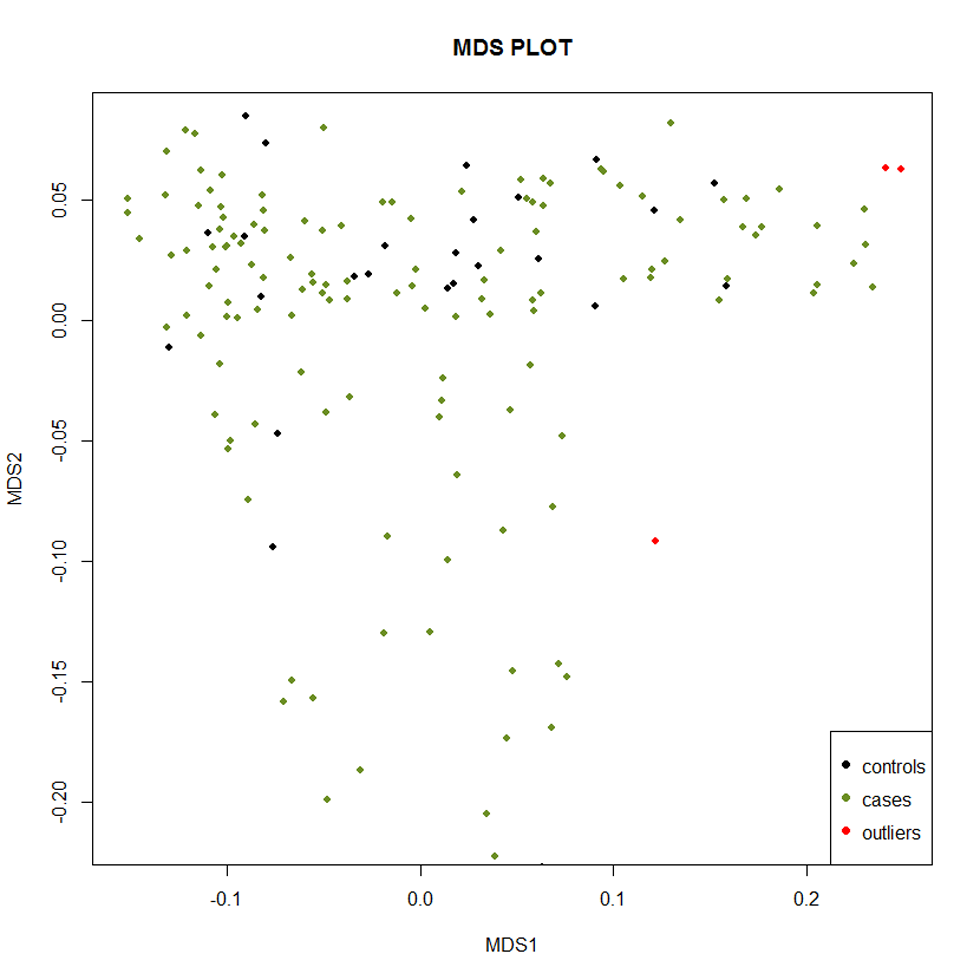

Supplement: Figure S1 — Multidimensional scaling (MDS) plot showing the genomic kinships between the analyzed Labrador Retrievers. This plot visualizes the overall genetic distances between the dogs based on 2,000 markers randomly selected out of the total of 113,021 SNP markers. Cases and controls do not form separate clusters, which is an essential prerequisite for a successful GWAS. (TIF) [file pone.0039176.s001.tif]
